# The effect of brain-computer interface controlled functional electrical stimulation training on rehabilitation of upper limb after stroke: a systematic review and meta-analysis

**DOI:** 10.3389/fnhum.2024.1438095

**Published:** 2024-09-26

**Authors:** Chunlin Ren, Xinmin Li, Qian Gao, Mengyang Pan, Jing Wang, Fangjie Yang, Zhenfei Duan, Pengxue Guo, Yasu Zhang

**Affiliations:** ^1^Rehabilitation Medicine College, Henan University of Chinese Medicine, Zhengzhou, China; ^2^School of Traditional Chinese Medicine, Henan University of Chinese Medicine, Zhengzhou, China; ^3^School of Exercise and Health, Shanghai University of Sport, Shanghai, China

**Keywords:** stroke, brain-computer interface, functional electrical stimulation, upper limb function, meta-analysis

## Abstract

**Introduction:**

Several clinical studies have demonstrated that brain-computer interfaces (BCIs) controlled functional electrical stimulation (FES) facilitate neurological recovery in patients with stroke. This review aims to evaluate the effectiveness of BCI-FES training on upper limb functional recovery in stroke patients.

**Methods:**

PubMed, Embase, Cochrane Library, Science Direct and Web of Science were systematically searched from inception to October 2023. Randomized controlled trials (RCTs) employing BCI-FES training were included. The methodological quality of the RCTs was assessed using the PEDro scale. Meta-analysis was conducted using RevMan 5.4.1 and STATA 18.

**Results:**

The meta-analysis comprised 290 patients from 10 RCTs. Results showed a moderate effect size in upper limb function recovery through BCI-FES training (SMD = 0.50, 95% CI: 0.26–0.73, I^2^ = 0%, *p* < 0.0001). Subgroup analysis revealed that BCI-FES training significantly enhanced upper limb motor function in BCI-FES vs. FES group (SMD = 0.37, 95% CI: 0.00–0.74, I^2^ = 21%, *p* = 0.05), and the BCI-FES + CR vs. CR group (SMD = 0.61, 95% CI: 0.28–0.95, I^2^ = 0%, *p* = 0.0003). Moreover, BCI-FES training demonstrated effectiveness in both subacute (SMD = 0.56, 95% CI: 0.25–0.87, I^2^ = 0%, *p* = 0.0004) and chronic groups (SMD = 0.42, 95% CI: 0.05–0.78, I^2^ = 45%, *p* = 0.02). Subgroup analysis showed that both adjusting (SMD = 0.55, 95% CI: 0.24–0.87, I^2^ = 0%, *p* = 0.0006) and fixing (SMD = 0.43, 95% CI: 0.07–0.78, I^2^ = 46%, *p* = 0.02). BCI thresholds before training significantly improved motor function in stroke patients. Both motor imagery (MI) (SMD = 0.41 95% CI: 0.12–0.71, I^2^ = 13%, *p* = 0.006) and action observation (AO) (SMD = 0.73, 95% CI: 0.26–1.20, I^2^ = 0%, *p* = 0.002) as mental tasks significantly improved upper limb function in stroke patients.

**Discussion:**

BCI-FES has significant immediate effects on upper limb function in subacute and chronic stroke patients, but evidence for its long-term impact remains limited. Using AO as the mental task may be a more effective BCI-FES training strategy.

**Systematic review registration:**

Identifier: CRD42023485744, https://www.crd.york.ac.uk/prospero/display_record.php?ID=CRD42023485744.

## 1 Introduction

Over the past three decades, there has been a significant increase in stroke incidence and mortality rates (GBD 2019 Stroke Collaborators, 2021). Approximately 80% of stroke patients experience upper limb dysfunction, greatly impacting daily activities and limiting social participation (Zhang et al., 2020; Feigin, 2008). Upper limb recovery is a fundamental aspect of stroke rehabilitation and is crucial for mitigating disability (Pollock et al., 2014). Technological advancements in neuroscience research have expanded rehabilitation options. Brain-computer interfaces (BCIs), transcranial magnetic stimulation, and robotic training have emerged as potential approaches for post-stroke rehabilitation (Vink et al., 2023; Zhang et al., 2022; Takebayashi et al., 2022; Frisoli et al., 2022; Guo et al., 2022; Caria et al., 2020). Numerous studies have confirmed that BCI-based training significantly improves upper limb motor function in stroke patients and the combination of BCI with functional electrical stimulation (BCI-FES) may be more effective (Nojima et al., 2022; Peng et al., 2022; Bai et al., 2022).

The BCI-FES system typically consists of a BCI unit, a BCI-FES interface assembly and an FES module (Khan et al., 2023). Pre-programmed therapeutic sessions are tailored to target specific movements for stroke patients, and researchers will adjust the BCI threshold based on patient performance. Within these sessions, patients are instructed to engage in motor imagery (MI) or action observation (AO), both of which effectively induce Electroencephalogram (EEG) changes. Subsequently, through training and decoding algorithms, these EEG signals are matched to specific imagined or observed movements, enabling control of the FES. Finally, the FES device delivers electrical stimulation to the affected muscles to perform the desired movement. Compared to other BCI training systems, BCI-FES directly stimulates muscle movement through the FES, which simultaneously provides proprioceptive feedback. This sensory input plays a vital role in neurorehabilitation by promoting neural reorganization (Remsik et al., 2022).

In 2009, Daly et al. first demonstrated the feasibility of BCI-FES for treating upper limb motor impairments in stroke patients (Daly et al., 2009). Since then, numerous innovations have broadened the applicability and effectiveness of BCI-FES systems in stroke rehabilitation. Rehtian-Romagosa and colleagues designed a recoveriX system, which integrates BCI-FES with virtual reality (Sebastián-Romagosa et al., 2020). This integration highlights the potential for combining BCI-FES with innovative technologies to further improve rehabilitation outcomes. Zhang et al. developed an adaptive BCI-FES system that dynamically adjusts task difficulty based on stroke severity, demonstrating the promise of personalized treatment strategies (Zhang et al., 2023). In future research, it may be feasible to customize specific BCI-FES systems based on the different stages and severities of stroke. Personalized services can be provided by understanding individual needs and capabilities.

Although BCI-FES training shows promise for enhancing motor function recovery in stroke patients, its widespread clinical application lacks substantial evidence (Simon et al., 2021). Variations in BCI-FES training strategies across studies, such as mental tasks, BCI threshold adjustments and intervention duration, have posed challenges in establishing its clinical efficacy. Therefore, this study aims to assess the immediate efficacy of BCI-FES training in improving upper limb recovery in stroke patients and to explore the impact of adjusting the BCI threshold and employing different mental tasks on its effectiveness. Additionally, the study used meta-regression analysis to explore factors that may influence effect size. The findings can provide valuable insights for refining treatment strategies.

## 2 Materials and methods

We followed the PRISMA guidelines for reporting systematic literature review and meta-analysis (Moher et al., 2009). The protocol has been registered on PROSPERO (CRD42023485744).

### 2.1 Data sources and search strategy

A comprehensive literature search of PubMed, Embase, Cochrane Library, Science Direct, and Web of Science was conducted from inception through October 2023. The keywords included “brain-computer interface OR brain-machine interface OR BCI OR BMI” and “stroke OR cerebral infarction OR cerebral hemorrhage OR cerebral vascular accident,” Additionally, we conducted a manual search by screening the reference lists of previous systematic reviews and meta-analysis to identify additional relevant articles for inclusion in our analysis.

### 2.2 Inclusion and exclusion criteria

The systematic review focused on studies in English, using the PICOS framework to set the inclusion criteria:

Participants: Adults (≥18 years) who had experienced a stroke, regardless of clinical variables like gender, stroke severity, nationality, or education level.

Intervention: The experimental group received BCI-FES training.

Comparison: The control group received sham BCI-FES training, conventional rehabilitation, or no intervention.

Outcomes: At least one outcome measure related to upper limb motor function was reported.

Study Design: Randomized controlled trials (RCTs).

Exclusion criteria comprised: (1) Duplicate studies. (2) Non-RCTs. (3) Used BCI-FES training in both intervention and control groups. (4) Studies with unavailable full texts or missing relevant outcome data.

### 2.3 Data extraction

Two reviewers independently evaluated the titles and abstracts of the retrieved articles, followed by assessment of the full-text articles. Data from these articles were then validated and extracted based on predetermined criteria, including study characteristics, participant profiles, interventions, control interventions, outcome measures, intervention dosages, BCI threshold adjustment, and types of mental tasks. Post-intervention assessments were collected as parameters for analysis of the immediate clinical effects.

### 2.4 Quality assessment

Two independent evaluators conducted a comprehensive assessment of the methodological quality of the controlled studies using the Physiotherapy Evidence Database (PEDro) scale (Moseley et al., 2002). To ensure consistency, any discrepancies between the evaluators were resolved through discussion and, when necessary, consultation with a third independent reviewer. After a thorough examination of the studies, the evaluators reached a unanimous decision. The PEDro scale comprises 11 criteria, which encompass various factors, including randomization, blinding, dropout rates, intention to treat, allocation concealment, and data reporting. These criteria are used to assess potential bias in clinical trials. Each of the remaining 10 criteria is awarded one point if met by the study except for the first criterion. The cumulative score is determined by summing these points. Studies scoring 9–10 on the PEDro scale are categorized as “excellent” quality, 6–8 as “good” quality, 4–5 as “fair” quality, and those scoring below 4 are deemed “poor” quality (Foley et al., 2003). We performed the quality assessment strictly according to the PEDro assessment guidelines.^1^

### 2.5 Data analysis

The Fugl-Meyer Motor Function of Upper Extremity Scale (FMA-UE) is a widely recognized stroke-specific impairment index with excellent properties (Hernández et al., 2019). However, one study employed the Manual Function Test as the primary outcome measure (Jang et al., 2016). To include this measure in our meta-analysis, we utilized the standardized mean difference (SMD) and 95% confidence interval (CI) as the pooled effect size estimators. The effect size was determined from the mean post-treatment scores of the experimental and control groups, along with a 95% confidence interval. Statistical significance was defined as *p*-values <0.05. Effect sizes were classified as large (SMD = 0.8), medium (SMD = 0.5) and small (SMD = 0.2). In instances where mean or standard deviation data were absent in the selected paper, we either contacted the corresponding author or computed them using available data. Heterogeneity was evaluated using the I^2^ statistic, with I^2^ ≥ 50% indicating significant heterogeneity. If I^2^ was less than 50%, the fixed effects model was applied; otherwise, the random effects model was used (Borenstein et al., 2010). We conducted sensitivity analysis using two approaches to assess the robustness of our meta-analysis: by including only high-quality studies, and by sequentially removing one study. Egger’s test was used to quantify publication bias. Subgroup analysis was performed to investigate the impact of variables such as the stroke stage, BCI threshold adjustment, and different mental tasks. Univariate meta-regression analysis was performed to explore associations between baseline patient age, session duration, cumulative training time and effect size. All data analysis were executed using RevMan 5.4.1 and STATA 18.

## 3 Results

### 3.1 Screening process and results of studies

**Figure 1:**
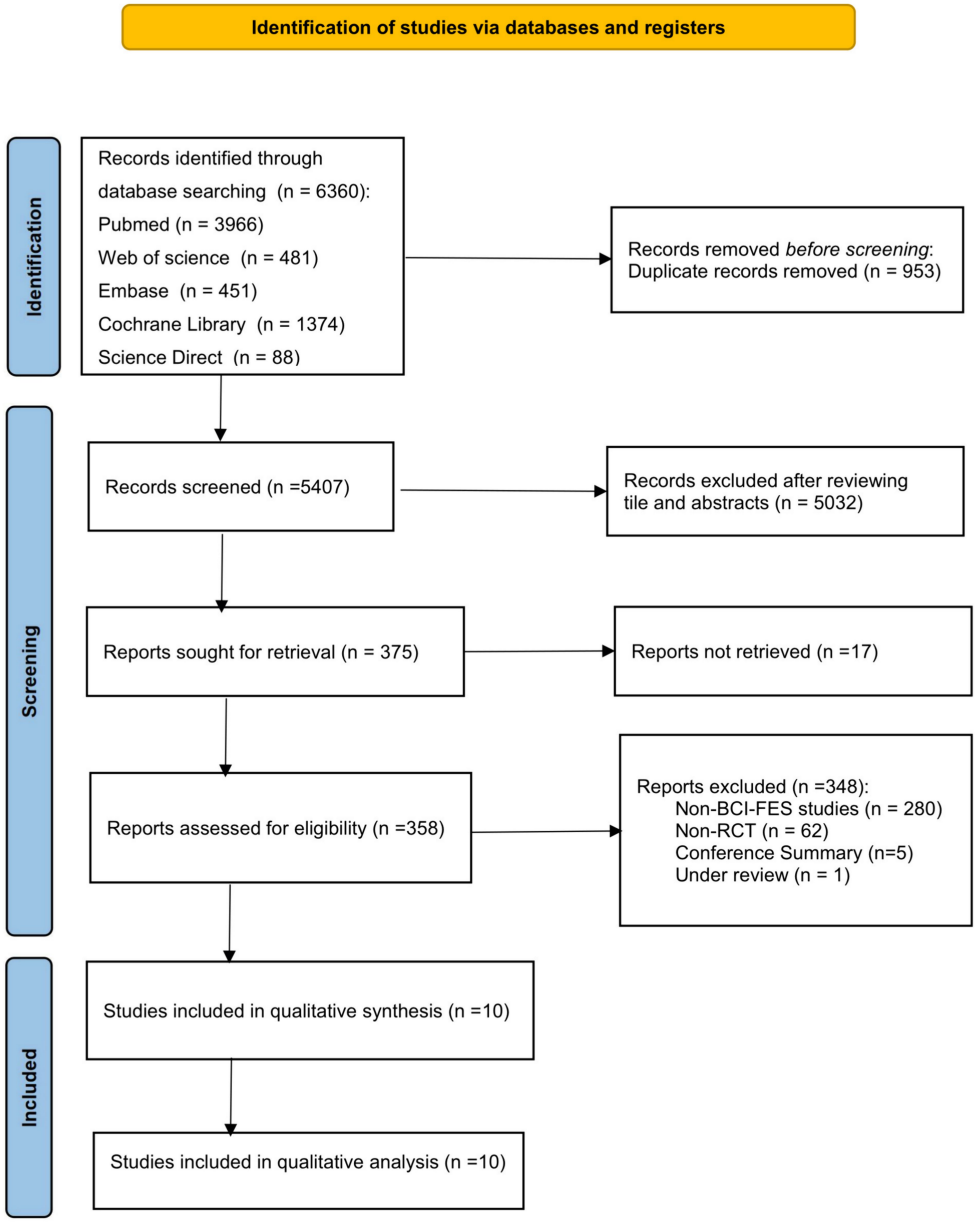
Flow chart of study select.

After retrieving 6,360 articles from 5 database and eliminating 953 duplicates, 5,407 records underwent screening based on title and author names. Finally, 10 articles meeting the selection criteria were included in this meta-analysis. The screening process is depicted in Figure 1.

### 3.2 Characteristics of the included studies

The characteristics of RCTs are summarized in Table 1. The meta-analysis involved 290 patients from 10 studies. Among these, 5 studies focused on chronic patients (Biasiucci et al., 2018; Miao et al., 2020; Lee et al., 2020; Kim et al., 2015; Zhan et al., 2022), 5 on subacute patients (Zhang et al., 2023; Jang et al., 2016; Li et al., 2014, Liu et al., 2023, Liao et al., 2023). In 5 of the 10 studies, researchers adjusted the BCI threshold before each treatment session (Biasiucci et al., 2018; Zhang et al., 2023; Liu et al., 2023; Lee et al., 2020; Li et al., 2014), while in the remaining 5 studies, the BCI threshold was fixed (Jang et al., 2016; Miao et al., 2020; Kim et al., 2015, Zhan et al., 2022, Liao et al., 2023). Six studies utilized MI (Jang et al., 2016; Liu et al., 2023; Miao et al., 2020; Li et al., 2014, Zhan et al., 2022, Liao et al., 2023), 3 used AO (Jang et al., 2016; Lee et al., 2020; Kim et al., 2015), and 1 employed motor attempt (Biasiucci et al., 2018) as mental tasks in BCI-FES training. Treatment sessions ranged from 20 to 60 min, with total treatment duration varying from 4 to 24 h. Among the 10 studies included, 2 were rated as excellent (Biasiucci et al., 2018; Zhang et al., 2023), 4 as good (Liu et al., 2023; Lee et al., 2020; Kim et al., 2015; Li et al., 2014, Liao et al., 2023), and 3 as fair in quality (Jang et al., 2016; Miao et al., 2020; Zhan et al., 2022). The PEDro scores ranged from 5 to 10, with an average score of 7.00 (1.76). The methodological quality of the included studies is detailed in Table 2.

**Table 1:** Characteristics of the included studies.

Study	N		Age (years)		Time from stroke		Intervention	Control	BCI threshold	Mental task	Intervention time	Outcome measures
	E	C	E	C	E	C						
Liu et al. (2023)	30	30	52.50 (45.0, 59.3)	53.00 (38.5, 59.50)	≤1 mo	≤1 mo	BCI-FES + CR	CR	Adjusted	MI	20 min/d, 5d/wk., 3wk	FMA-UE, MBI, WMFT, NIHSS
Zhang et al. (2023)	17	16	53.20 (7.9)	49.3 (13.5)	2.6 (1.3) mo	3.4 (1.5) mo	BCI-FES	FES	Adjusted	MI	40 min/d, 5d/wk., 4wk	FMA-UE, MMT, ROM
Liao et al. (2023)	20	20	61.5 (3.8)	61.0 (3.7)	20.1 (2.2) d	20.6 (2.4) d	BCI-FES + CR	CR	Fixed	MI	60 min/d, 5d/wk., 3wk	FMA-UE, MAS
Zhan et al. (2022)	12	12	72.5 (6.92)	77.08 (5.57)	36.67 (15.03) mo	40.58 (29.93) mo	BCI-FES	FES	Fixed	MI	40 min/d 3d/wk., 4wk	FMA-UE
Lee et al. (2020)	13	13	55.15 (11.57)	58.30 (9.19)	7.46 (1.61) mo	8.30 (1.97) mo	BCI-FES	FES	Adjusted	AO	30 min/d, 5d/wk., 4wk	FMA-UE, MBI, WMFT, MAL
Miao et al. (2020)	8	8	48.80 (16.70)	50.30 (17.1)	18.3 (10.90) mo	11.1 (5.0) mo	BCI-FES + CR	CR	Fixed	MI	20 min/d 3d/wk., 4wk	FMA-UE
Biasiucci et al. (2018)	14	13	56.36 (9.91)	59.00 (12.36)	39.79 (45.90) mo	33.46 (30.51) mo	BCI-FES	Sham BCI-FES	Adjusted	MA	60 min/d, 2 d/wk., 5wk	FMA-UE, MAS, ESS
Jang et al. (2016)	10	10	61.10 (13.77)	61.70 (12.09)	4.40 (0.97) mo	4.10 (0.74) mo	BCI-FES	FES	Fixed	AO	20 min/d, 5 d/wk., 6wk	MFT, VAS, MAS
Kim et al. (2015)	15	15	59.09 (8.07)	59.93 (9.79)	8.27 (1.98) mo	7.80 (1.78) mo	BCI-FES + CR	CR	Fixed	AO	30 min/d, 5d/wk., 4wk	FMA-UE, MAL, MBI, ROM
Li et al. (2014)	7	7	66.3 (4.9)	67.10 (6.0)	2. 2 (1. 8) mo	2. 8 (2. 0) mo	BCI-FES	FES	Adjusted	MI	60 min/d, 3d/wk., 8wk	FMA-UE, ARAT

E, Experimental group; C, Control group; BCI, Brain-computer interface FES functional electrical stimulation; CR, Conventional Rehabilitation; min, minute(s); h, hour(s); d, day(s); wk., week(s); mo, month(s). FMA, Fugl-Meyer assessment; UE, Upper extremity; ARAT, Action research arm test; WMFT, Wolf Motor function test MAS Modified Ashworth scale; VAS, visual analog scale; MAL, Motor activity log; NIHSS, National Institute of Health Stroke Scale; MBI, Modified Barthel Index; ROM, Range of motion; MMT, manual muscle testing; ESS, European Stroke scale; EEG, electroencephalography; MFT, Manual Function Test.

**Table 2:** Methodological quality assessment of the controlled studies.

PEDro items												
Study	1	2	3	4	5	6	7	8	9	10	11	Total
Liu 2023	1	1	1	1			1		1	1	1	7
Zhang 2023	1	1	1	1	1	1	1	1	1	1	1	10
Liao 2023	1	1		1			1		1	1	1	6
Zhan 2022	1	1		1					1	1	1	5
Miao 2020	1	1	1	1					1		1	5
Lee 2020	1	1	1	1			1	1	1	1	1	8
Biasuicci2018	1	1	1	1	1		1	1	1	1	1	9
Jang 2016	1	1		1			1		1		1	5
Kim 2015	1	1	1	1			1		1	1	1	8
Li 2014	1	1	1	1				1	1	1	1	7

1 = eligibility criteria; 2 = random allocation; 3 = concealed allocation; 4 = baseline comparability; 5 = blind subjects; 6 = blind therapists; 7 = blind assessors; 8 = adequate follow-up; 9 = intention-to-treat analysis; 10 = between-group comparisons; 11 = point estimates and variability.

### 3.3 The effects of BCI-FES training on upper limb function

**Figure 2:**
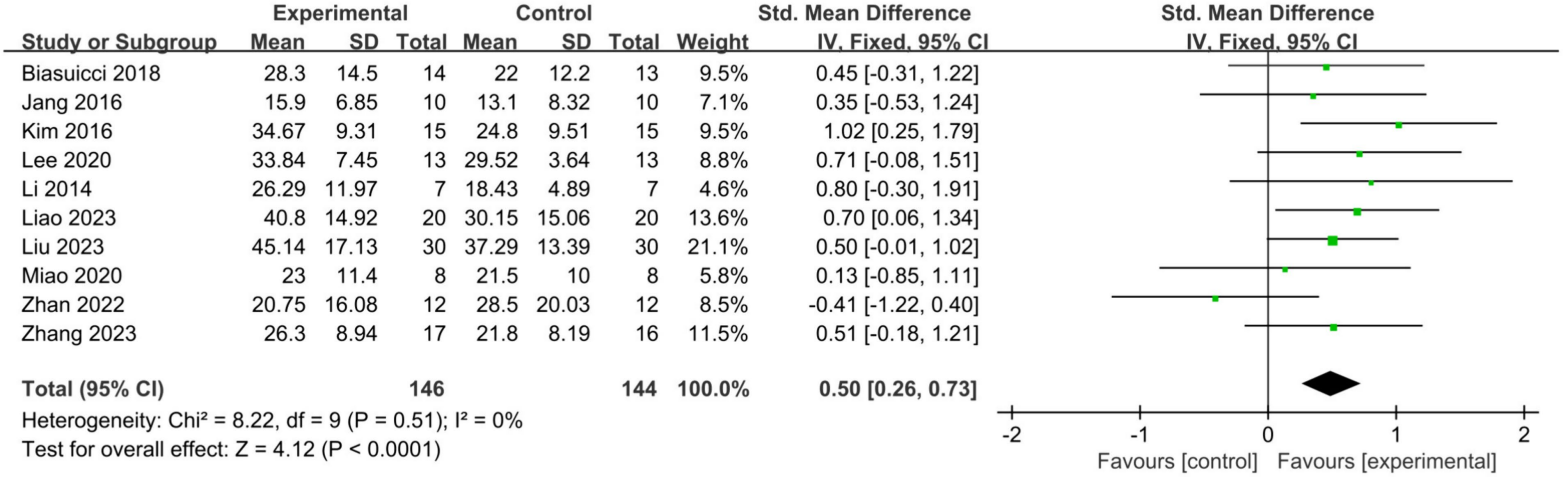
Comparison of the effects of immediate effects of the BCI-FES group and control group on the upper limb recovery in stroke patients.

**Figure 3:**
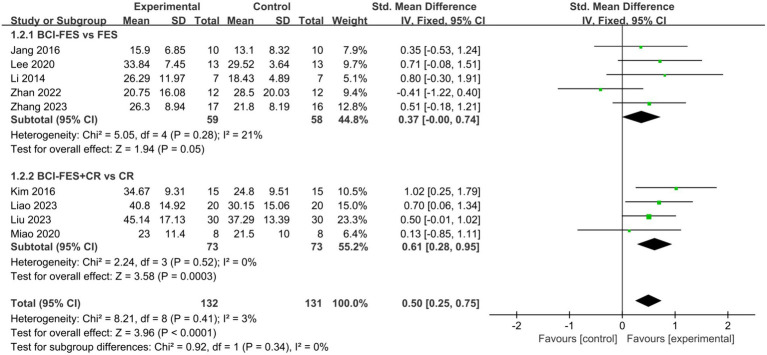
Comparison of the effects of BCI-FES vs. FES and BCI-FES + CR vs. CR on upper Limb recovery in stroke patients.

Across the 10 studies, 146 patients were allocated to the BCI-FES group, while 144 patients were assigned to the control group. Pooled results indicated that BCI-FES training (SMD = 0.50, 95% CI: 0.26–0.73, I^2^ = 0%, *p* < 0.0001) significantly enhanced upper limb motor function compared to the control intervention (Figure 2). No evidence of publication bias was observed according to the Egger’s test (*β* = −0.625; se = 1.529; *p* = 0.693). In the sensitivity analysis, we sequentially removed each study and reassessed the pooled results. It was observed that both heterogeneity and significance varied only within a narrow range. When only studies of good methodological quality or higher (PEDro score > 5) were included, the effect of BCI-FES training on upper limb motor function increased (SMD = 0.64, 95% CI: 0.37–0.90, I^2^ = 0%, *p* < 0.0001) (Figure 3). The Egger’s test also showed no evidence of publication bias (β = 1.146, se = 0.936, *p* = 0.276).

### 3.4 Subgroup analysis

#### 3.4.1 Different rehabilitation groups

To explore the effects of BCI-FES interventions across different control groups, we categorized the studies into two subgroups: BCI-FES vs. FES and BCI-FES + CR vs. CR. Subgroup analysis revealed that BCI-FES training significantly enhanced upper limb motor function in BCI-FES vs. FES group (SMD = 0.37, 95% CI: 0.00–0.74, I^2^ = 21%, *p* = 0.05), and the BCI-FES + CR vs. CR group (SMD = 0.61, 95% CI: 0.28–0.95, I^2^ = 0%, *p* = 0.0003) (Figure 3). The difference between the two subgroups was not statistically significant (*p* = 0.34). When only studies of good methodological quality and higher were included, both groups remained significant with increased effect sizes: BCI-FES vs. FES group (SMD = 0.64, 95% CI: 0.16–1.11, I^2^ = 0%, *p* = 0.008) and the BCI-FES + CR vs. CR group (SMD = 0.67, 95% CI: 0.32–1.03, I^2^ = 0%, *p* = 0.0002). There was no statistically significant difference between the two groups (*p* = 0.90).

#### 3.4.2 Stroke stages

**Figure 4:**
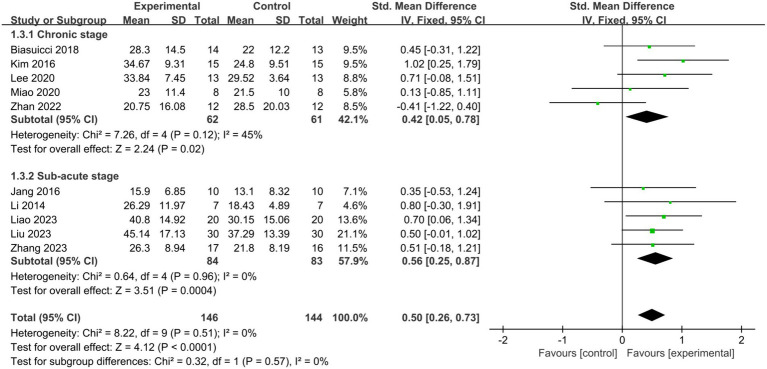
Comparison of the effects of BCI-FES training among patients in different stages of stroke.

To explore variations in the effectiveness of BCI-FES training among patients at different stages of stroke. Patients within 6 months post-stroke were classified as subacute, while those beyond 6 months were classified as chronic. Subgroup analysis revealed that BCI-FES training significantly enhanced upper limb motor function in both the chronic group (SMD = 0.42, 95% CI: 0.05–0.78, I^2^ = 45%, *p* = 0.02) and the subacute group (SMD = 0.56, 95% CI: 0.25–0.87, I^2^ = 0%, *p* = 0.0004), with no statistically significant difference between the two groups (*p* = 0.57) (Figure 4). When only studies of good methodological quality and higher were included, both groups remained significant with increased effect sizes: the chronic group (SMD = 0.73, 95% CI: 0.28–1.18, I^2^ = 0%, *p* = 0.001) and the subacute group (SMD = 0.58, 95% CI: 0.25–0.92, I^2^ = 0%, *p* = 0.0006). There was no statistically significant difference between the two groups (*p* = 0.61).

#### 3.4.3 BCI threshold adjustment or fixation

**Figure 5:**
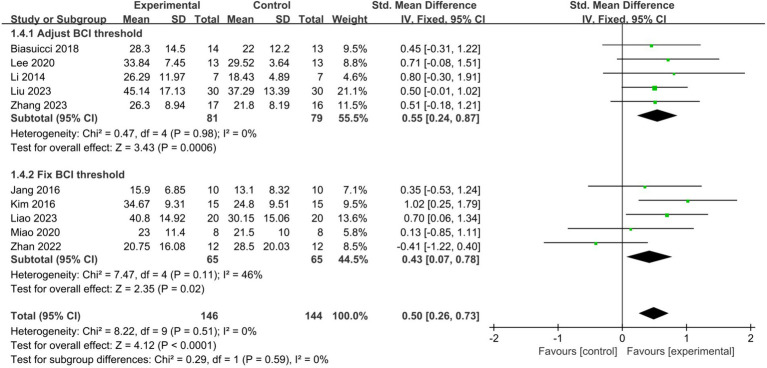
Comparison of the impact of adjusting the threshold before each treatment session or fixing it on upper limb functional recovery in stroke patients.

Among the 10 studies included in our analysis, 5 adjusted the BCI threshold before each treatment session, while the remaining 5 maintained a fixed threshold. Subgroup analyses showed that both adjusting the BCI threshold before training (SMD = 0.55, 95% CI: 0.24–0.87, I^2^ = 0%, *p* = 0.0006) and maintaining a fixed BCI threshold (SMD = 0.43, 95% CI: 0.07–0.78, I^2^ = 46%, *p* = 0.02) significantly improved upper limb motor function in stroke patients. There was no statistically significant difference between the two approaches (*p* = 0.59) (Figure 5). All three studies of fair quality were in the group that maintained a fixed BCI threshold. When only studies of good methodological quality and higher were included, the fixed BCI threshold group remained significant, with an increased effect size (SMD = 0.83, 95% CI: 0.34–1.32, I^2^ = 0%, *p* = 0.0009). There was no statistically significant difference between the two approaches (*p* = 0.36).

#### 3.4.4 Different mental tasks

**Figure 6:**
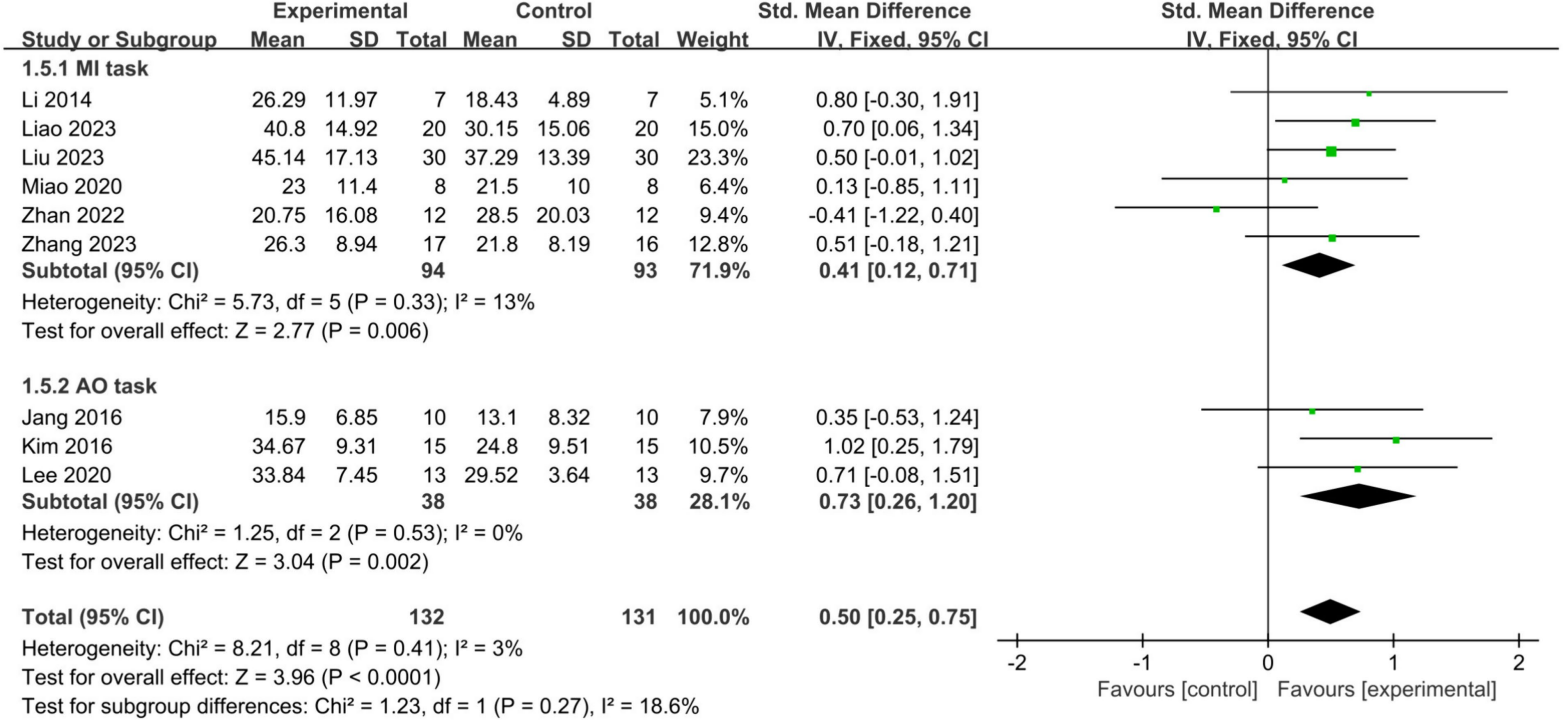
Comparison of the effects of the MI tasks group and AO tasks group on the upper limb in stroke patients.

MI and AO represent the two primary mental tasks utilized in BCI-FES training. Subgroup analysis demonstrated that employing MI (SMD = 0.41, 95% CI: 0.12–0.71, I^2^ = 13%, *p* = 0.006) or AO (SMD = 0.73, 95% CI: 0.26–1.20, I^2^ = 0%, *p* = 0.002) significantly improved upper limb motor function in stroke patients, with no statistically significant difference observed between the two methods (*p* = 0.27) (Figure 6). When only studies of good methodological quality and higher were included, both groups remained significant with increased effect sizes: the MI group (SMD = 0.54, 95% CI: 0.16–0.93, I^2^ = 0%, *p* = 0.006) and the AO group (SMD = 0.87, 95% CI: 0.32–1.43, I^2^ = 0%, *p* = 0.002). There was no statistically significant difference between the two tasks (*p* = 0.34).

### 3.5 Meta-regression

To assess the potential influence of other clinical factors, we performed meta-regression analysis on baseline patient age and the duration of interventions. Single-factor analysis revealed that baseline patient age (coefficient = 0.0159, se = 0.1822, *p* = 0.384), session duration (coefficient = 0.0026, se = 0.0075, *p* = 0.733), and cumulative training time (coefficient = 0.0004, se = 0.0004, *p* = 0.271) were not significant predictors of effect size.

## 4 Discussion

This study aims to evaluate the immediate effects of BCI-FES training on upper limb recovery in stroke patients and to analyze the impact of BCI threshold adjustments and different mental tasks on its effectiveness. Additionally, meta-regression analysis was performed to identify factors that may influence the overall effect size. These findings may provide valuable insights for optimizing BCI-FES treatment strategies in stroke rehabilitation.

The quality of the included studies was assessed using the PEDro scale, a validated tool widely employed in rehabilitation and physical therapy research to evaluate the methodological rigor of randomized controlled trials. The 10 studies included had an average score of 7.00 (1.76), indicating generally good methodological quality. The meta-analysis of 10 studies demonstrated that BCI-FES training had a favorable medium effect on upper limb function (SMD = 0.50). Heterogeneity analysis and sensitivity analysis confirmed the robustness of the result. When restricting the meta analysis to studies with good methodological quality and higher, the effect of BCI-FES training increased (SMD = 0.64). No heterogeneity was obversed after excluding studies of fair quality (I^2^ = 0) and Egger’s test showed no evidence of publication bias (*p* = 0.276). This suggested that the 3 fair-quality studies slightly underestimated the effect size. Similarly, in all subgroup analysis, limiting the analysis to good quality and higher studies consistently showed that BCI-FES remained effective in improving upper limb motor function, with increased effect sizes. In conclusion, although variations in study quality have influence on the effect sizes, they do not significantly affect the overall assessment of BCI-FES effectiveness in improving upper limb motor function in stroke patients.

The adjustment of the BCI threshold aims to improve the accuracy of mental tasks, which has been shown to be associated with better functional recovery (Myrden and Chau, 2015). Among the 10 studies included, nearly all studies with good and higher quality implemented BCI threshold adjustments. This suggests that researchers recognized the link between BCI threshold adjustments and improved clinical outcomes. However, subgroup analysis revealed that no statistically significant difference between the two approaches. Further studies are required to validate these findings and to clarify the specific role of BCI threshold adjustments in enhancing upper extremity functional recovery in stroke patients.

AO and MI serve as primary mental tasks capable of eliciting EEG signal alterations during BCI-FES training. The subgroup analysis demonstrated that both AO and MI, when used as mental tasks during BCI-FES training, significantly enhance upper limb motor function in stroke patients. However, AO appears to yield greater improvements in upper limb function compared to MI. AO involves activating the brain’s motor areas by observing specific movements performed by others. Based on mirror neuron theory, observing such movements can trigger the activation of the neural network associated with them (Garrison et al., 2013). Conversely, MI entails mentally simulating movements to activate the brain’s motor areas without physical execution (Jackson et al., 2001). Neuroimaging studies have extensively explored the neural substrates of MI, revealing significant overlap with the neural network responsible for motor execution (Hétu et al., 2013). However, approximately 20–30% of stroke patients, due to impaired sensory-motor areas or advanced age, fail to produce Event-Related Desynchronization through MI (Tani et al., 2018; Ahn, 2013). Research by Allison and Neuper reported that even in healthy individuals, around 15–30% fail to produce distinct ERD (Allison and Neuper, 2010). Furthermore, studies by Kübler et al. have shown that despite several months of MI training, performance improvements for certain participants remain limited (Kübler et al., 1999). In contrast to MI, AO does not necessitate initial skill proficiency; even passive observation by individuals with limited proficiency can activate the brain’s motor areas (Binks et al., 2023). MI may be suboptimal for stroke patients learning complex movements beyond their repertoire. Further research is warranted to directly compare the roles of these two mental tasks in BCI-FES training.

In the meta-regression analysis, baseline age was not identified as a significant predictor of effect size. This suggests that BCI-FES provides similar benefits across different age groups. Session duration and cumulative training time were also not significant predictors of effect size. These findings are similar to previous studies (Nojima et al., 2022; Bai et al., 2022). We speculate that the effectiveness of BCI-FES training may rely more on the accuracy of mental task. As reported by Miao et al., stroke patients with the highest mental task accuracy achieved the greatest improvements in upper limb function (Miao et al., 2020).

Based on previous studies, two hypotheses were proposed regarding the mechanisms by which BCI-FES training facilitates the recovery of motor function after stroke: enhancing the excitability of the ipsilateral M1 and rectifying interhemispheric imbalance. Numerous studies have shown that BCI-FES training not only significantly enhances connections between neurons in existing neural pathways but also fosters the formation of new neural connections. These developments facilitate the activation of both the motor cortex and PM in the lesioned hemisphere (Min et al., 2020). Functionally, BCI-FES training induces noteworthy changes in cortical activation patterns. Initially, there is substantial activation observed in the contralateral motor cortex post-stroke. As recovery progresses, activation in the motor cortex of the lesioned hemisphere gradually intensifies, signifying the process of rectifying hemispheric imbalance (Ramos-Murguialday et al., 2013).

Increased excitability within the ipsilesional motor cortex is considered symbol of motor rehabilitation in the affected upper limb. Studies by Biasiucci et al. and Zhang et al. observed heightened connectivity between the ipsilesional primary M1 and PM following training (Biasiucci et al., 2018; Zhang et al., 2023). Additionally, these studies indicated varied *μ*, *α*, and beta rhythm activity across brain regions, with notable activation of μ and beta rhythms in the M1 (Zhang et al., 2023; Liu et al., 2023; Lee et al., 2020). Numerous pre-and post-single-group design studies with healthy subjects have also demonstrated that BCI significantly activates the prefrontal cortex, PMC, and posterior parietal cortex. This increased connectivity and rhythmic activity in ipsilesional areas hold significant implications for motor rehabilitation (Zhang et al., 2014; Nicolo et al., 2015; Schulz, 2012). In summary, BCI-FES training may enhance connectivity along existing neural pathways, facilitate the formation of new neural connections, and promote Hebbian-like plasticity through synchronized activation of cerebral motor areas and peripheral effectors (Behboodi et al., 2022).

Another mechanism involves addressing interhemispheric imbalances. Miao et al. and Li et al. conducted a study examining changes in patients’ brain topography before and after BCI-FES training. Their findings suggested that during BCI-FES training, cortical activation initially spreads across broad areas before concentrating in motor or adjacent cortical areas in the lesioned hemisphere (Miao et al., 2020; Li et al., 2014). Initially, due to significant brain function impairment, there was a notable increase in activation in motor areas bilaterally, particularly in the non-lesioned hemisphere, which is crucial for stroke recovery (Serrien et al., 2004; Rehme et al., 2011; Ward et al., 2003). However, sustained activation of the contralateral prefrontal and parietal cortex may indicate slower and incomplete recovery (Murphy and Corbett, 2009). Over time, activation in the non-lesioned hemisphere diminishes, and neural activation gradually shifts back to the diseased hemisphere, suggesting the correction of interhemispheric imbalances (Park et al., 2011; Markram et al., 2011). However, a review has highlighted the lack of compelling evidence to support the existence of interhemispheric inhibitory imbalance (McDonnell and Stinear, 2017). Caution is warranted when considering the repair of interhemispheric imbalance as a mechanism for BCI-FES training to promote stroke rehabilitation.

## 5 Limitations

The limitation of this study is the small sample size, which may affect the reliability of the results. Although we employed various methods to explore heterogeneity, differences in study methodologies could still have a potential impact on the findings. Additionally, previous clinical trials have rarely addressed the long-term follow-up effects of BCI-FES training. Future research should focus on increasing sample sizes and conducting extended follow-up to more accurately assess the clinical value of BCI-FES training.

## 6 Conclusion

BCI-FES training has a significant immediate impact on enhancing upper limb function in both subacute and chronic stroke patients. Adjusting the threshold before each BCI-FES session did not result in significantly better upper limb motor function recovery compared to fixing the threshold. Using the AO as mental task may be a better BCI-FES training strategy. However, future larger trials are needed to validate these results and explore the long-term sustained effects of BCI-FES.

## Data Availability

The original contributions presented in the study are included in the article/supplementary material, further inquiries can be directed to the corresponding author.

## Footnotes

^1^ https://pedro.org.au/
